# Immunosuppressive Treatment for Myocarditis in the Pediatric Population: A Meta-Analysis

**DOI:** 10.3389/fped.2019.00430

**Published:** 2019-11-15

**Authors:** Bing He, Xiaoou Li, Dan Li

**Affiliations:** Department of Pediatrics, Renmin Hospital of Wuhan University, Wuhan, China

**Keywords:** immunosuppressive treatment, myocarditis, pediatric, cardiac function, meta-analysis

## Abstract

The use of immunosuppressants in the treatment of myocarditis in children remains controversial. The aim of this meta-analysis is to summarize the current empirical evidence for immunosuppressive treatment for myocarditis in the pediatric population. We searched PubMed, MEDLINE, and Embase for articles to identify studies analyzing the efficiency of immunosuppressive treatment in the pediatric population. Pooled estimates were generated using fixed- or random-effect models. Heterogeneity within studies was assessed using Cochran's *Q* and *I*^2^ statistics. Funnel plots and Begg's rank correlation method were constructed to evaluate publication bias. Sensitivity analyses were also conducted to evaluate the potential sources of heterogeneity. After a detailed screening of 159 studies, six separate studies were identified, with 181 patients in the immunosuppressive treatment group, and 199 in the conventional treatment group. The immunosuppressive treatment group showed a significant improvement in left ventricular ejection fraction (LVEF) [mean difference 1.10; 95% CI: 0.41, 1.79] and significantly decreased left ventricular end-diastolic dimension (LVEDD) [mean difference −0.77 mm, 95% CI: −1.35 to −0.20 mm] when compared to the conventional treatment group. Furthermore, the risk of death and heart transplant in conventional treatment was significantly higher than in the immunosuppressive treatment group [relative risk (RR): 4.74; 95% CI: 2.69, 8.35]. No significant heterogeneity across the studies was observed. There was no evidence of publication bias when assessed by Begg's test.

**Conclusions:** There may be a possible benefit, in the short term, to the addition of immunosuppressive therapy in the management of myocarditis in the pediatric population. However, further prospective investigation is warranted to validate this finding.

## Introduction

Acute myocarditis is an inflammatory cardiac disease in children. Acute myocarditis is common in the developing countries (1), with about 20% of children admitted to hospital with heart failure due to acute myocarditis (2).

The mortality of myocarditis was reported to be about 23–50% (3). The pathogenesis of viral myocarditis is now recognized to have three distinct phases (4). Among the three distinct phases, the autoimmune phase is believed to play a major role in the pathogenesis of viral myocarditis, and the use of immunosuppressive agents may be useful in myocarditis.

Currently, according to the guidelines of the European Society of Cardiology (ESC), immunosuppression treatment is not routinely recommended for myocarditis in the pediatric population (5). Immunosuppression treatment seems to be effective in the adult population, but it may not be suited for the pediatric population. Furthermore, the pathophysiology and etiology of myocarditis and the response to immunosuppression treatment in the pediatric population may be significantly different from that of the adult population. So, the use of immunosuppressants in the treatment of myocarditis in children remains controversial. However, despite the lack of large randomized controlled trials (RCTs), benefits have been suggested in some reports (6–11).

To our knowledge, a comprehensive and systematic meta-analysis of studies investigating the efficacy of immunosuppressive treatment in the pediatric population with acute myocarditis has not been performed. This report presents just such a systematic review and meta-analysis.

## Materials and Methods

### Search Strategy

PubMed, MEDLINE, and Embase were searched for articles written in the English language that assessed the interactions between myocarditis and immunosuppressive treatment using the keywords “myocarditis” OR “carditis” AND “immunosuppressive treatment” AND “children” OR “pediatric,” up to July 20th, 2018. Reference lists of included studies and relevant reviews were also manually searched to identify remaining studies.

### Selection Criteria

In our meta-analysis, we included both RCTs and case–control studies (CCTs) that compared immunosuppressive treatment with conventional treatment in patients with myocarditis. The diagnosis of myocarditis was made by histological, immunologic, and immunohistochemical criteria (12) or clinically by the investigators.

Immunosuppressive treatment included the use of prednisone, azathioprine, cyclosporine, and intravenous immunoglobulin G (IVIG).

Eligible studies had at least one of two outcome measures: cardiovascular improvement and survival. The following parameters were used as markers of improvement in cardiovascular status: (1) resolution of symptoms of congestive cardiac failure; (2) hemodynamic measurements using cardiac catheterization, two-dimensional echocardiography, or radionuclide scans; and (3) resolution of histological findings on repeat endomyocardial biopsy (EMB) (defined by a decrease in, or an absence of, inflammatory infiltrate in the myocardium or by the Dallas criteria). Patients were considered to have survived if they were alive and had avoided a heart transplant at the end of the study period as defined by each individual study. Those who died or required a heart transplant were considered non-survivors. Exclusion criteria were as follows: (1) research subjects were not children; (2) lack of outcome reports such as death or improvement in cardiovascular parameters [such as left ventricular ejection fraction (LVEF)]; (4) lack of conventional treatment for control group; (4) animal experiments, case reports, commentaries, or multiple papers from the same study; and (5) studies with no original data were not included. For studies without enough quantitative data, the correspondent author was contacted, and if no answer was obtained, the studies were excluded.

This meta-analysis aimed to focus on randomized controlled trials (RCTs), but due to the scarcity of existing published data, non-RCTs, retrospective studies, and larger case series with conventional treatment for the control group were considered. Isolated case reports and case series with fewer than four subjects were excluded. Furthermore, the estimate of the treatment effect based on an unduly small sample size is likely to be imprecise.

### Quality Assessment

For studies included in this meta-analysis containing RCTs and non-RCTs [prospective non-controlled trial (PNCT), CCT], it is hard to do quality assessment using one method. So the quality assessments of RCTs and non-RCTs were separated. For RCTs, the *Cochrane Handbook for Systematic Reviews of Interventions Version 5.1.0* (13) was used to judge the studies' quality, including random sequence generation, allocation concealment, blinding of participants and personnel, blinding of outcome assessment, incomplete outcome data, and selective reporting. For non-RCTs, the Newcastle–Ottawa Scale (NOS) (14) was used to judge the studies' quality. The three broad perspectives included the following: selection of the study population, comparability between groups, and assessment of the exposure or outcome. We evaluated seven items as follows: (1) representative of the study population; (2) description of the study methods; (3) selection of controls; (4) definition of controls; (5) clearly defined the immunosuppressive agent; (6) assessment of outcome; (7) reasonable follow-up length more than 1 year (to assess outcome).

### Statistics

Continuous variables are reported as mean value ± standard deviation, and categorical variables are presented as relative risk (RR) and 95% confidence interval (CI). Heterogeneity within studies was assessed using Cochran's Q and *I*^2^ statistics. If the *P*-value for heterogeneity was determined to be <0.05 or the *I*^2^ value was >50%, the presence of heterogeneity was taken into consideration. When heterogeneity was significant (*P* < 0.05 or *I*^2^ > 50%), the random-effects model was applied; otherwise, the fixed-effects model was used. Funnel plots and Begg's rank correlation method were constructed to evaluate publication bias. Sensitivity analysis was performed to assess the influence of each individual study on the estimated effects by omitting of individual studies. According to the *Cochrane Handbook for Systematic Reviews of Interventions Version 5.1.0*, the subgroups could be combined into a single group; the changed standard deviation from baseline that was not reported directly in articles could be calculated by formula. Data from all studies were pooled using STATA software (version 12.0, STATA Corporation, College Station, TX).

## Results

### Eligible Studies

**Figure 1:**
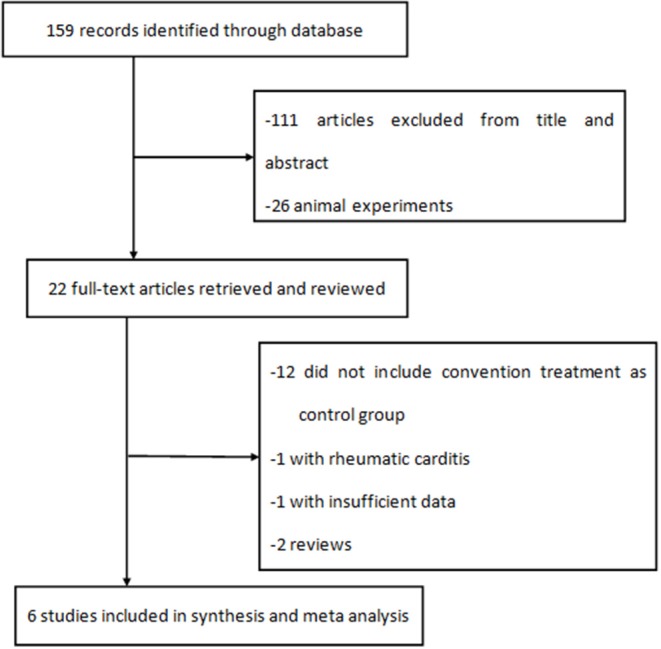
Flowchart of search strategy and selection of studies for meta-analysis.

A total of 159 literature reports were initially identified. After screening the titles and abstracts, 22 full-text articles were assessed for eligibility, and six were included in the analysis (Figure 1). One of six studies was an RCT, two of six were PNCTs, and the other three were CCTs.

### Characteristics of Studies

There were 380 pediatric patients enrolled in six studies, with 181 patients in the immunosuppressive treatment group and 199 in the conventional treatment group. The details of the study characteristics are shown in Table 1. The immunosuppressive agents included prednisone, azathioprine, cyclosporine, and IVIG. The mean age in the studies ranged from 8 months to 15 years. The mean follow-up period in the studies ranged from 8 months to 7 years.

**Table 1:** Characteristics of studies included in the meta-analysis.

**Study**	**N**	**Age**	**Study methodology**	**IMSA**	**IMSA dosage, time of IMSA start**	**Follow-up**	**Observed variables**	**Inclusion criteria**
Camargo et al. (9)	50	5 months−15 years	PNCT	P, CyA	P & A: 2.5 mg/kg/d, 1 week; 2.0 mg/kg/d, 3 weeks; 1.5 mg/kg/d, 4 weeks Cy: 1.5 mg/kg/d, 1 week; 1.0 mg/kg/d, 7 weeks; 0.5 mg/kg/d, 1 week	8.4 ± 1.2 months	LVEDD, LVEF, PWP, CI, HR	Active myocarditis based on EMB findings
Aziz et al. (6)	68	3.7 ± 2.9 years	RCT	P	2 mg/kg/d, 1 month	15.1 ± 9.2 months	LVEDD, LVESD, LVEF	Duration of symptoms for <3 months and continued LV failure and reduced EF
Drucker et al. (7)	46	–	CCT	IVIG	2,000 mg/kg 24 h; 1,000 mg/kg/d, 1 weeks	10.5 ± 2.1 months	LVFS, LVEDD, death	Acute (<3 months) onset of congestive heart failure and echocardiographic documentation of diminished LV function and EMB
Bhatt et al. (8)	83	4.4 ± 3.2 years	PNCT	IVIG	400 mg/kg/d, 5 days	-	LVEF, death	Had viral infection with fever of <2 weeks' duration; developed acute and severe heart failure after this illness; evidence of LV dysfunction on echocardiography EF <40%; no previous or family history of cardiomyopathy
Gagliardi et al. (10)	114	36.6 ± 42.8 months	CCT	P, Cy	P: 2 mg/kg/d, 1 month; 0.5 mg/kg/d, 6 months; Cy: 6–8 mg/kg/d until blood concentration reached 170–210 ng/cm^3^	13 years	LVEF, LVEDV, death	Congestive heart failure patients received right cardiac characterization and EMB
Camargo et al. (11)	10	42.1 ± 18.9 months	CCT	P, A	2.5 mg/kg, 4 weeks; 1.5 mg/kg, 4 weeks (both drugs)	9 months	LVEF, CI, death	Patients presenting with dilated cardiomyopathy who were clinically stable, under ambulatory care, with LVEF between 15 and 30%

*PNCT, prospective non-controlled trial; RCT, randomized controlled trial; CCT, case–control study (including historical controls); IMSA, immunosuppressive agent; P, prednisolone; CyA, cyclosporine; A, azathioprine; IVIG, intravenous immunoglobulin G; LVEF, left ventricular ejection fraction; LVEDD, left ventricular diastolic dimension diameter; LVESD, left ventricular systolic dimension diameter; PWP, pulmonary wedge pressure; HR, heart rate; LVFS, left ventricular fractional shortening; CI, cardiac index, EMB, endomyocardial biopsy*.

For those diagnoses proven by EMB, the histologic type is important because therapy and prognosis are different between giant cell myocarditis, eosinophilic myocarditis, lymphocytic myocarditis, and so on. However, we couldn't indicate the exact histologic type, because these studies didn't provide these data. Camargo et al. (9) reported that due to technical reasons, EMB was detected in only 19 patients. Positive histologic evolution was found in 1 of 4 in the control group compared to 12 of 15 in the immunosuppressive treatment group. Camargo et al. (11) reported three enteroviruses, one adenovirus, and one cytomegalovirus in EMB detection. Drucker et al. (7) reported that positive biopsy was found in 12 of 19 patients in the IVIG group and 8 of 20 in the non-IVIG group. Gagliardi et al. (10) didn't provide the exact histologic type and just reported that giant cells were not seen in any of the EMBs.

### Quality Assessment of Included Studies

The quality assessment of one RCT study was according to the *Cochrane Handbook for Systematic Reviews of Interventions Version 5.1.0*. The RCT study demonstrated low risk of bias in selection bias, performance bias, attrition bias, and reporting bias, and an unclear risk of bias in detection bias. Five non-RCT studies (7–11) were evaluated on seven quality aspects based on the NOS; an overview of the quality assessment is listed in Table 2. Only one (7) of five studies met all the criteria for quality assessment. All studies provided a representative of the study population, description of study methods, and selection of controls, and clearly defined the immunosuppressive agent and assessment of outcome. Only one study (10) did not provide the definition of controls. Only two studies (7, 10) had a long follow-up period (≥1 year).

**Table 2:** Quality assessment of non-RCTs.

**Study**	**Representative of the study population**	**Description** **of study methods**	**Selection of controls**	**Definition of controls**	**Clearly defined** **the immunosuppressive agent**	**Assessment** **of outcome**	**Follow-up length more than 1 year**
Camargo et al. (9)	√	√	√	√	√	√	
Drucker et al. (7)	√	√	√	√	√	√	√
Bhatt et al. (8)	√	√	√	√	√	√	
Gagliardi et al. (10)	√	√	√		√	√	√
Camargo et al. (11)	√	√	√	√	√	√	

### Immunosuppressive Treatment and LVEF

**Figure 2:**
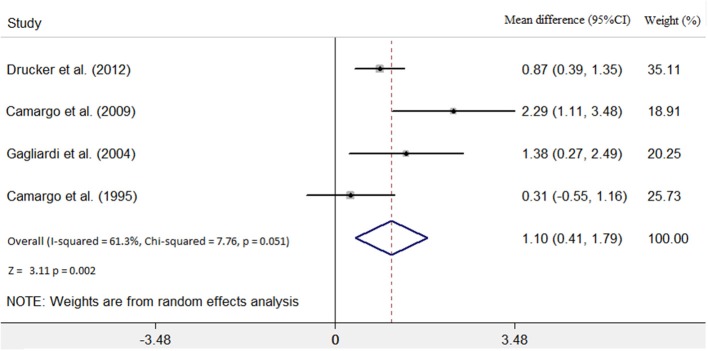
Immunosuppressive treatment vs. conventional treatment on the outcome of left ventricular ejection fraction (LVEF) in the pediatric population with acute myocarditis.

Four studies (7, 9–11) identified the relationship between the immunosuppressive treatment and LVEF change. A total of 140 patients were enrolled, 56 patients in the immunosuppressive treatment group, and 84 patients in the conventional treatment group. Compared with the control group, LVEF increased (mean difference 1.10; 95% CI: 0.41, 1.79) significantly in the immunosuppressive treatment group. There was significant heterogeneity among these studies (Q test; *I*^2^ = 61.3%, *p* = 0.051) (Figure 2). A random-effects model was used. After excluding one relatively short-follow-up-duration study (9), no significant heterogeneity across the studies was observed (*I*^2^ = 18.1%, *p* = 0.300). There was no evidence of publication bias when assessed by Begg's test (*p* = 0.479; Table 3).

**Table 3:** Results of publication bias (Egger Test).

**Group**	***N***	***t***	***P***	**95% CI**
LVEF	4	0.86	0.479	−7.86, 11.82
LVEDD	3	4.49	0.140	−22.63, 47.34
Death/heart transplant	4	1.57	0.256	−2.92, 6.29

### Immunosuppressive Treatment and LVEDD

**Figure 3:**
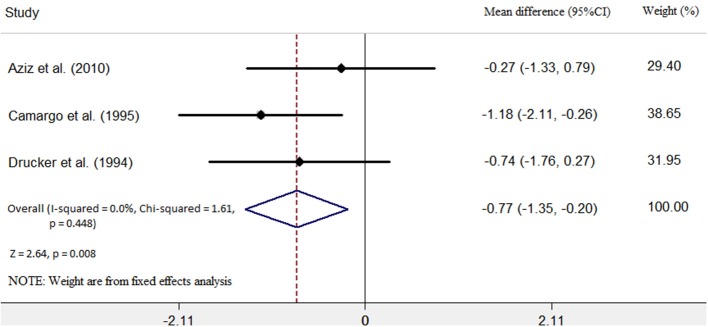
Immunosuppressive treatment vs. conventional treatment on the outcome of left ventricular end-diastolic diameter (LVEDD) in the pediatric population with acute myocarditis.

Three studies (6, 7, 9) identified the relationship between the immunosuppressive treatment and left ventricular end-diastolic dimension (LVEDD) change. A total of 67 patients were enrolled, 25 patients in the immunosuppressive treatment group, and 42 patients in the conventional treatment group. Compared with the conventional treatment group, LVEDD decreased (mean difference −0.77 mm; 95% CI: −1.35, −0.20 mm) significantly in the immunosuppressive treatment group. There was no heterogeneity among these studies (Q test; *I*^2^ = 0.0%, *p* = 0.448) (Figure 3). A fixed-effects model was used. There was no evidence of publication bias when assessed by Begg's test (*p* = 0.140; Table 3).

### Immunosuppressive Treatment and Death and Transplantation

**Figure 4:**
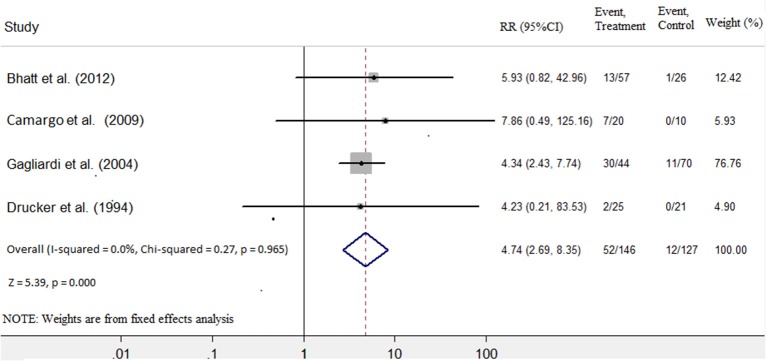
Immunosuppressive treatment vs. conventional treatment on the outcome of rate of death or transplantation in the pediatric population with acute myocarditis.

Four studies (7, 8, 10, 11) were identified that referred to death and heart transplantation as outcomes, with 127 patients in the immunosuppressive treatment group and 146 patients in the conventional treatment group. The rates of death or heart transplantation were 9.4% in the immunosuppressive treatment group and 35.6% in the conventional treatment group. The risk of death or heart transplantation in the conventional treatment group was significantly higher than in the immunosuppressive treatment group (RR: 4.74; 95% CI: 2.69, 8.35). The was no heterogeneity among these studies (Q test; *I*^2^ = 0.0%, *p* = 0.965) (Figure 4), and a fixed-effects model was used. There was no evidence of publication bias when assessed by Begg's test (*p* = 0.256; Table 3).

### Sensitivity Analyses

The sensitivity analysis results suggested that no individual studies significantly affected the pooled effect of the association between immunosuppressive treatment and the LVEF, LVEDD, and risk of death and heart transplantation, indicating our statistically robust results.

#### Endomyocardial Biopsy

**Figure 5:**
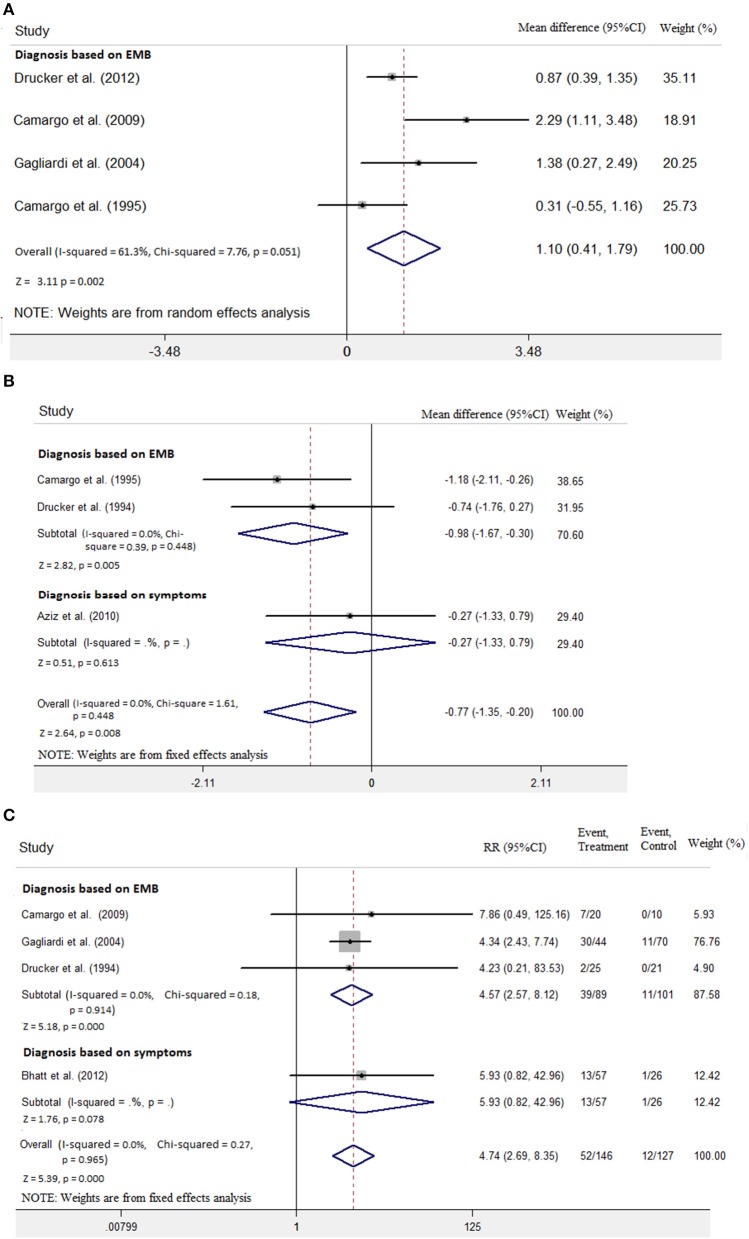
Diagnosis based on endomyocardial biopsy (EMB) vs. diagnosis based on symptoms on the outcome of **(A)** LVEF, **(B)** LVEDD, and **(C)** rate of death or transplantation in the pediatric population with acute myocarditis.

EMB is the gold standard for the diagnosis of myocarditis. We tried to do subanalysis separately for diagnosis using EMB and diagnosis based on symptoms and LVEF. Only three included studies diagnosed myocarditis with EMB (7, 9, 10). As shown in Figure 5A, LVEF increase was significantly higher in the group with diagnosis based on EMB (mean difference 1.10; 95% CI: 0.41, 1.79). LVEDD significantly decreased in the group with diagnosis based on EMB (mean difference −0.98, 95% CI: −1.67, −0.30 vs. mean difference −0.27, 95% CI: −1.33, 0.79; Figure 5B). Moreover, the risk of death or heart transplantation in the group with diagnosis based on EMB was significantly lower than in the diagnosis based on symptoms (RR: 4.57, 95% CI: 2.57, 8.12 vs. RR: 5.93, 95% CI: 0.82, 42.96; Figure 5C).

## Discussion

This meta-analysis focused on the efficiency of immunosuppressive treatment in the pediatric population with acute myocarditis. Our results identified a possible association between immunosuppressive treatment and increased LVEF, decreased LVEDD, and reduced risk of death and heart transplant in the pediatric population with myocarditis. This meta-analysis provided some evidence that immunosuppressive treatment in the short term might be effective in the improvement of heart function and survival. And immunosuppressive treatment based on EMB seems to be more effective than treatment based on symptoms in increasing LVEF, decreasing LVEDD, and reducing the risk of death and heart transplant in the pediatric population with myocarditis. However, the present review was an exploratory analysis because the numbers of the included studies are too small to make any meaningful inferences between immunosuppressive and IVIG therapy.

It was reported that acute myocarditis rarely occurs in the pediatric population (15). The incidence of myocarditis was difficult to determine and was reported to be about 0.15–0.6% in the overall population (15, 16). A study reported myocarditis representing 0.05% of all pediatric hospital discharges from birth through age 21 years (17). Another recent study of Finland showed that the overall incidence rate of myocarditis was 1.95/100,000 person-years (18). On the one hand, it is probably underestimated because of a considerable number of asymptomatic patients. On the other hand, the diagnosis of myocarditis is challenging because symptoms are frequently non-specific, especially in infants and children. Currently, the Dallas criteria (19) were used to make a definite diagnosis worldwide. Although EMB is the gold standard for the diagnosis of myocarditis, it is an invasive procedure; current guidelines recommend EMB only in a limited number of clinical scenarios that do not include some common presentations of myocarditis (20). Recently, the role of cardiac MRI (CMR) in the diagnosis of myocarditis has increased. However, because CMR cannot exclude viral forms of myocarditis, the use of CMR is limited (21).

Myocardial damage in myocarditis is mediated partly by immunological mechanisms. Viral infections are the most common causes of myocarditis in children (22), but subsequent myocardial damage appears to be mediated by autoimmune mechanisms in addition to direct viral infection (23). Animal data suggested a role for autoimmunity alone or secondary to viral infection in the damage to cardiac myocytes, most likely as a result of myocyte necrosis (24) and subsequent release of self-antigens previously hidden to the immune system (25).

According to the pathogenesis of myocarditis, immunosuppressive treatment would be theoretically useful in improving the prognosis of myocarditis, and immunosuppression treatment has been used in pediatric myocarditis over a few decades. Numerous studies reported good outcomes with immunosuppressive therapy. However, the ESC statement currently recommends consideration of immunosuppression in proven autoimmune forms of myocarditis due to the absence of multicenter RCTs (5). In this meta-analysis, we concluded that immunosuppressive treatment might be effective in the improvement of heart function and survival in myocarditis children, although only one RCT was included.

Furthermore, IVIG has both anti-viral and immunomodulatory effects; thus, studies using these agents should be separately analyzed from those using “pure” immunosuppressive agents, such as steroids and azathioprine. Meta-analysis was not possible, because only two relevant studies (7, 8) were found. The cardiac function in the IVIG treatment group was more improved than that in the conventional treatment group (LVEF: 16.7 ± 4.1 vs. 2.7 ± 1.6, *p* < 0.05). The rates of death or transplantation in the immunosuppressive treatment group were significantly lower than 18.3% in the conventional treatment group (2.1 vs. 18.3%, *p* < 0.05). Moreover, we analyzed the effect of IVIG on the rates of death or transplantation, compared with pure immunosuppressive agents, such as steroids and azathioprine; the rates of death or transplantation in the IVIG group seem lower (2.2 vs. 15.7%). In the absence of multicenter RCTs in EMB-proven myocarditis of viral or autoimmune origin, we should be very cautious when using IVIG. More randomized studies focused on IVIG treatment in the pediatric population with myocarditis are needed in the future.

This meta-analysis shows new insights that an autoimmune mechanism is largely responsible in the pathogenesis of acute myocarditis and perhaps has at least a theoretical role for immunosuppression in pediatric patients. However, the results still need to be confirmed by larger multicenter randomized studies in the future.

### Limitations

The included studies had small sample sizes, and only six studies are included in the present study, of which only one is an RCT study; this might result in a lack of statistical power to detect a significant difference in the treatment effect. Moreover, we were able to ascertain publication bias in only four of six studies, which means only four of six studies' data could be merged, which may have impacted the analysis of the findings of this meta-analysis. Due to the included studies' lack of long-term follow-up (only two studies had median follow-up > 1 year), their inferences can only be applied to short-term outcomes. In this meta-analysis, we couldn't provide exact data of viral genome and histologic type, because the included studies didn't report these data even if this information was important to the therapy and prognosis. RCTs in the future should pay more attention to the viral genome and histologic type data. In addition, we tried to do subanalysis related to RCTs on the efficiency of immunosuppressive treatment in the pediatric population. Meta-analysis was not possible, because only one relevant study was found. Although the results confirm a good outcome of the immunosuppressive treatment, the results seem to be not so feasible and should be interpreted cautiously.

## Conclusions

The present meta-analysis suggests that immunosuppressive treatment in the short term may significantly improve LVEF, reduce LVEDD, and reduce the risk of death and heart transplant in pediatric population with myocarditis. Although this meta-analysis reported beneficial outcomes with immunosuppressive therapy, the results have to be interpreted cautiously because only one RCT was included in this meta-analysis; more large-scale RCTs are required in the future.

## Author Contributions

BH is responsible for the provision of the overall idea and writing articles. XL collects data and modifies the paper. DL is responsible for statistical analysis.

### Conflict of Interest

The authors declare that the research was conducted in the absence of any commercial or financial relationships that could be construed as a potential conflict of interest.
